# Successful treatment of a keratoacanthoma in a young patient with the application of topical 5% imiquimod cream

**DOI:** 10.1002/ccr3.4920

**Published:** 2021-11-06

**Authors:** Kerasia‐Maria Plachouri, Francesk Mulita, Dimitrios Bousis, Levan Tchabashvili, Elias Liolis, Charalampos Kaplanis, Ioannis Perdikaris, Fotios Iliopoulos, Georgios‐Ioannis Verras, Vasilios Tolias, Sophia Georgiou

**Affiliations:** ^1^ Department of Dermatology General University Hospital of Patras Patras Greece; ^2^ Department of General Surgery General University Hospital of Patras Patras Greece; ^3^ Department of Internal Medicine General University Hospital of Patras Patras Greece

**Keywords:** epithelial tumor, imiquimod cream, keratoacanthomas

## Abstract

Keratoacanthomas (KA) are epithelial tumors that present as rapidly evolving nodules with a central hyperkeratotic plug and occasionally show signs of spontaneous regression. A 21‐year‐old patient strongly refused the diagnostic biopsy and insisted on a nonsurgical treatment. He was successfully treated with imiquimod 5% cream.

## Graphical abstract

Surgical excision is the most common treatment option for keratoacanthomas. However, the topical application of the imiquimod 5% cream is a safe and effective option, especially for patients who refuse surgical removal.

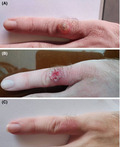

## 1 CASE DESCRIPTION

**FIGURE 1:**
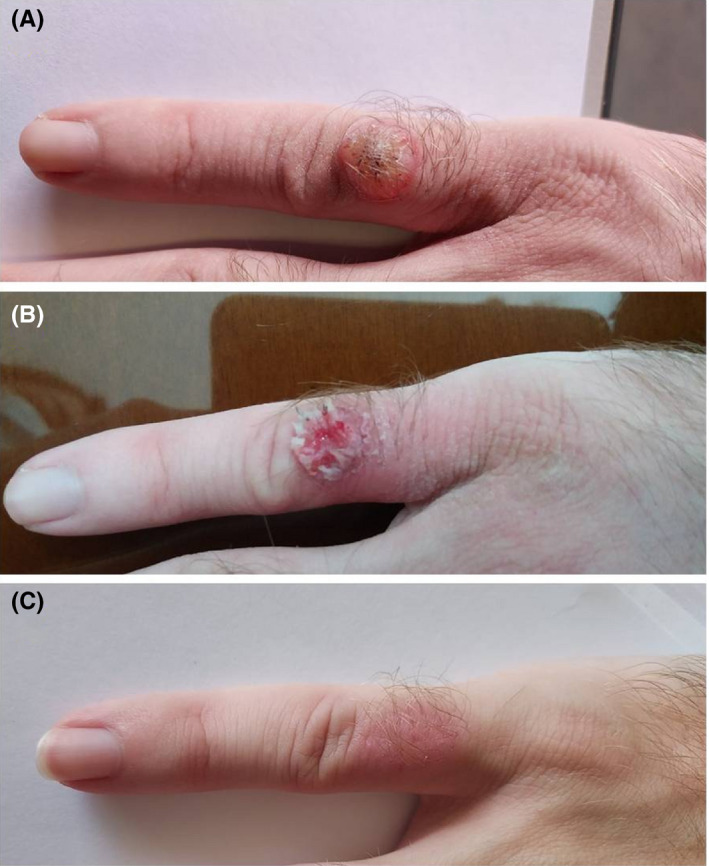
A. KA in the proximal fifth digit of the right hand, before treatment B. KA in the proximal fifth digit of the right hand, 3 weeks after treatment initiation C. Complete resolution of the KA in the proximal fifth digit of the right hand, 4 weeks after the completion of treatment

A 21‐year‐old man presented in our department due to an asymptomatic nodule in the proximal fifth digit of the right hand, that had first appeared 3 months prior to the referral. The lesion rapidly progressed in size during the first 4 weeks and afterward remained stable. The clinical examination revealed a firm skin‐colored nodule, of approximately 1.8 × 1.8 cm in size, with a central non‐removable keratinous plug (Figure 1A). The clinical diagnosis of keratoacanthoma was made, and a biopsy was recommended for diagnosis confirmation. The patient strongly refused the diagnostic biopsy and insisted on a nonsurgical treatment. We, therefore, suggested a regimen of topical imiquimod 5% cream under occlusion, for five consecutive days per week, over a period of 4 weeks. Two weeks after treatment initiation, a prominent local inflammatory reaction could be documented, resulting in crust formation and erosion (Figure 1B). Four weeks after the completion of treatment, the lesion was markedly flattened, until complete resolution was achieved (Figure 1C).

Keratoacanthomas (KA) are epithelial tumors that present as rapidly evolving nodules with a central hyperkeratotic plug and occasionally show signs of spontaneous regression.^1^ The treatment of choice for KAs is surgical excision, as it provides the advantage of complete tumor removal in a short period of time.^1^, ^2^ However, conservative therapeutic strategies are also to be considered.^2^

## CONFLICTS OF INTEREST

There are no conflicts of interest to declare.

## AUTHOR CONTRIBUTIONS

FM, EL, DB, LT, G‐IV, IP, FI, CK, VT, and K‐MP contributed to the clinical data collection and prepared the case report. FM, SG, and K‐MP contributed to the design of the case report presentation and performed the final revision of the manuscript.

## CONSENT

The written informed consent was obtained from the patient for publication of this case report.

## Data Availability

Data are available on request from the authors.
